# The Progress of Our School: Has It Been What It Should Have Been? If Not, Why Not?

**Published:** 1899-05

**Authors:** Edward Adams

**Affiliations:** Toronto, Canada


### PROCEEDINGS OF THE SOCIETY OF HOMŒOPATHICIANS,

### BUREAU OF CLINICAL MEDICINE.

F. W. PATCH, *Chairman.*

### THE PROGRESS OF OUR SCHOOL: HAS IT BEEN WHAT IT SHOULD HAVE BEEN? IF NOT, WHY NOT?

EDWARD ADAMS, M. D., TORONTO, CANADA.

MR. CHAIRMAN AND FELLOW-MEMBERS:

It will possibly occur to you that I have tackled a rather large subject—undertaken too great a contract—if so, you agree with me—so that it is with a strong feeling of hesitancy—a hesitancy extending over years past, and sufficiently strong to keep me silent while hoping that some other, with like feelings, though better fitted, would take the lead. However, I have waited in vain, and now, trusting that "the day of small things will not be despised," I hesitate no longer, but proceed to ask my questions and to try to answer them.

Some may suggest that I have "applied to the wrong court;" that my subject, pertaining neither to "homœopathic philosophy" nor to "clinical cases," is unsuitable for presentation to this Society of Homœopathicians, but I beg to differ, for if homœopathic societies, professing as their *raison d'etre* "the good and advancement of Homœopathy," do not take an interest in and action upon this and similar urgent questions, I know not where to apply.

Stock-taking is a prime necessity in all business undertakings, but I feel and fear that we as a school of medicine have neglected this duty, have been content to go on in a hap-hazard manner, "each for himself," knowing that he had "a good thing" and content that it should extend its blessings to himself and his immediate patrons.

But as "stock-taking" is a necessity in all successful business, so my plea for it in connection with our school may prove of benefit.

Mind you, I do not assert that such will be the case, nor even that with a thorough "stock-taking" we may not "jog along" as for the past hundred years, compelled to follow, or, better, content to follow methods—or the lack of methods—which in this year of 1898 A. D. and the 143d of Homœopathy, or since Hahnemann first expounded and proved the truth of our law, finds our school in a secondary position to the dominant school, finds us unrecognized by governments, general or State, except in a few instances, which depend upon or result from the homœopathic experience of the ruler or governor, and not upon an imperative demand from the people, *for truth*, and in big doses. As far as I can learn and judge, the promise of rapid growth anticipated by Hahnemann and his early disciples has not been realized.

To-day in most European countries, practitioners by this *truth*, this *law* are under a ban—have to defend their right to exist and practice medicine homœopathically—in fact, in defending themselves they have no time in which to spread the glad tidings of "a new and better way." In most countries outside of Europe our position is still worse—from a *very* few down to no representatives—and among the few we find too often that their professional existence depends upon their practicing Homœopathy surreptitiously, while among the people, as a whole, the title of homœopath—homœopathist—carries no greater weight, gives no greater information if as much, as "Indian doctor" or snake-charmer" did through this and the neighboring countries a few years ago.

This is not a state of affairs which should puff us up or encourage an indulgence in pride. Surely a vast field for missionary effort.

But to come nearer home. In Canada we are slowly advancing *backward*.

Some thirty years ago a medical council was created, solely through the weakness of our so-called leaders of that day, who for "a mess of pottage" sold our rights to an examining and licensing board. And the medical council became a Juggernaut, which has been slowly and surely, not alone preventing advance and progress, but crushing the life out of Homœopathy as a school. Having no homœopathic college on Canadian soil, our young men flocked to homœopathic colleges—so-called—in the United States, where after a two-year course of instruction, they returned home, where they were met by a strict examination in all subjects pertaining to medicine and surgery, including so-called homœopathic subjects, and a requirement of four full years spent in colleges and hospitals. This was bad enough for the newly fledged graduate anxious to benefit humanity, but when in addition the impression obtained, justly or unjustly, that no homœopathic graduate, known as such, would have a fair show, the obstacles became almost unsurmountable, but few being sufficiently certain of their ability and courageous enough to run the attendant risks, entered for the various examinations and succeeded in passing the same.

A few took advantage of an opening in the laws of the council, went to Great Britain or Ireland, obtained their license, under what they considered fairer conditions, where their leaning toward Homœopathy was unknown, and returning home, presented said license or degree and secured registration thereupon. Even at that time but few colleges in Great Britain and Ireland were recognized by *our* council, and since then this privilege, or loophole, as you may choose to regard it, has been cut off or closed entirely. The requirements in education and time spent in study have become more and more ironclad, so that now five full years must be spent in the pursuit of medical knowledge—the medicine of experience—divided as regards the medical council into a thorough matriculation examination, followed in due time by the junior, intermediate, and final examinations, which if passed successfully and certificates of five years' study being presented—the last of the five years having been spent in hospital and post-graduate work—the applicant is registered and granted a license, and his future professional acts receive the endorsement of law, *secundum artem*. No foreign physician, no matter how great his experience, how highly creditable his professional degrees, how high the position to which he has risen by his abilities, can be or will be permitetd to register or practice in Ontario, less even than the Dominion, for each Province has its monopoly, its close corporation in the shape of a medical council, without beginning anew and successfully negotiating all these above-mentioned examinations—an impossible task, as any physician of a few years' standing must admit. Do you wonder that many who would like to settle in Ontario are prevented by the above medical laws, and wisely decide that the price demanded is too great for the advantages offered? Scores of young men but lately graduated whose homes and interests were in Ontario, have settled in the United States, expatriated by too much law and red tape. Still these have not been lost to our school, and what Ontario has lost one or other of the States of your Union has gained. But these help to prove prophetic the utterance of an allopathic member of the council and an examiner for the council, who when the medical council became established and legal, exclaimed: "We have got those d—d homœopaths just where we want them. If we let them severely alone, they are bound to die out." And it looks as though the mantle of a Holmes had temporarily fallen upon him. I have been examiner in homœopathic subjects for two years, four examinations, and have had but one candidate, and he at the last, or May examination. I am glad to add that he was successful. But this represents about the average success for these many years past. The prospect for Homœopathy in Ontario and Canada as a whole cannot be called brilliant by the most optimistic. At the same time, our school and its practitioners in the United States may avoid our experience by turning their backs upon all allopathic offers to form a medical council of representatives of all schools of medicine; they invariably hold the balance of power. Distrust their offers, plead they "never so sweetly." "The voice may be the voice of Jacob, but the skin will be that of Esau," with all its psoric accumulations. Now as to your country, the only country in the world in which Homœopathy has made any showing worthy of mention, and yet when we come to compare the progress made with the facilities at hand, disappointment must result.

Homœopathy, like all truth, flourishes best in the midst of education and liberty. These she has found in the United States, hence the partial success, which though greater than in all other countries, leaves so much more yet to be accomplished, when we come to compare the number of its practitioners, their position in the eye of the law, or better, perhaps, of the Government, National and State. When we count the number of National or State institutions in which homœopathic truth and practice obtain, we are ready to exclaim with the ancient prophet, "How long, O Lord, how long shall my enemy be established against me?" Now, while admitting with thankfulness what has already been accomplished, I feel that the showing made is dwarfed by considerations of the country and its people, a country and a people whose watchwords are "Liberty, Freedom, Equality" (in the eye of the law!). Here, if anywhere on the footstool, under such favorable conditions, with such vast opportunities, you would expect to find that the "God-given truths of *pure* Homœopathy would find full and free acceptance," would run and be glorified.

Note my adjective, for it is important, *pure*. Pure Homœopathy.

But what do we find? Few even of those who believe in and patronize our system know anything about it, except possibly that cures result and the medicines prescribed are more palatable.

Under your Government, whose foundation principle *was* and should be, the equality of all men before the law, homœopathic professors and practitioners cannot hold professional office, unless their principles and name are held strictly *sub rosa*. We never hear of an avowed homœopathist—a fighting homœpath—being appointed a surgeon in your army or navy, nor even to the office of examiner of pensioners. And it is only of late years that any public institutions, and these under State control, and few in number both of States and institutions, are under homœopathic control. To my knowledge the Government of the United States in no way recognizes Homœopathy. If I can be successfully contradicted, no one will be better pleased than I to make the *amende*, and as a penance promise my next vote. Why, even to come down to the third great power (some assert it to be the first) in this great land, the corporations, monopolies, and companies forbid that a homœopathic physician, as such, should be appointed in their behalf. And so throughout the whole world and society at large Homœopathy does not occupy the position it should, and why? for all these remarks tend to one end, viz.: to discover the cause or causes, and as far as in me lies, aid in their removal. The task is great, but success, even partial, will richly reward honest and continuous effort. The history of the world can show no period so noted for advance in science and art, no period when the mind of man was more active in the search for truth, more ready to utilize when found; hence the absence of a universal appreciation and acceptance of the truth and blessing of Hahnemannian Homœopathy is the more exasperating.

To return to causes. I feel convinced that one of the principal causes is ignorance of homœopathic law and philosophy and lack of honesty in applying what is known by the vast majority of physicians calling themselves homœopaths. To judge from their practice such men should adopt the title of "eclectic." It is no easy task to follow Hahnemann's direction to take "the case correctly and from the totality of the symptoms select the indicated remedy and let it alone." How much easier to prescribe for a disease by name—to combat pain by tablets or pilules of Morphia—to meet the effects of malaria with massive doses of Quinine! But where is the Homœopathy in such practice? Allopathic physicians knowing of such practice, and knowing how different are the claims made for Homœopathy by pen and tongue, naturally exclaim, "Frauds," "dishonesty!" When they get bad cases they have to resort to our practice, and so, through the fault of some, who, though called by our name, are yet not of us, our school as a whole suffers, and those who know the truth, who live up to the law and practice by it, are alike condemned. Another illustration of the effects of ill associations, of being found in bad company. Of course my remarks refer to that large number who know in part but do not utilize even that partial knowledge conscientiously, and also to another large number who do not know and will not learn, who are content to be ignorant. There is likewise another section who err through ignorance and for whom some excuse can be made, viz.: those who have never been taught the truth.

In this year, A. D. 1898, it may be hard to believe that any great number of such so-called homœopathic physicians exist, and their influence in injuring the advance of Homœopathy is just as great as though no excuse could be found for them, and thus their excuse proves to be another great obstacle to homœopathic progress. Long ago was predicted the result of the "blind leading the blind," and in our school can be found a practical illustration in the excuse referred to above. I refer to our colleges which for many long years have been homœopathic only in name. The Faculties of these colleges are composed of men who either do not know the truth as it was in Hahnemann, or knowing it, keep the knowledge strictly to themselves.

For long years class after class of students have attended these institutions, the majority desirous of becoming homœopathic physicians, graduating and receiving diplomas which guaranteed to the world their ability to practice medicine according to homœopathic law, yet knowing nothing of Hahnemann and his writings, nor of his philosophy. They were no more prepared to reflect credit upon the school and advance the cause whose name they had assumed than if the homœopathic portion of the curriculum had been confined to a shilling reprint of some English domestic (homœopathic) practice. I speak feelingly, for I suffered and was wronged. Unfortunately I stood not alone, but had thousands of companions, graduates before, during, and since my college days. For all such I have ever felt the deepest sympathy, a sympathy which has broadened and deepened since I have learned something of homœopathic truth—something of the wonders—the *miracles*—which are performed by the practice of Homœopathy as taught by the master. Unfortunately, no matter how much of homœopathic truth a homœopathic physician obtains in after years, he never fully recovers from having been defrauded in his student days. Unless of the fortunate few who spent their *probation* in the office of a Hahnemannian homœopath, he is certain to have gotten into a rut, to have adopted methods lazy or careless, or both, certainly anything but the exactness his lately obtained knowledge demands and must and will have.

For all this he may thank his *Alma Mater;* and, as the effects of the wrongs from which he has suffered hampers his efforts, doubles his work and lessens his skill, a spirit of forgiveness for the wrong done, both of commission and omission, is not readily cultivated; in fact, I venture the assertion that, instead of forgiveness, his sense of the injury done him will become accentuated as he comes in contact with graduates of Hahnemannian homœopathic schools, of but a few years' existence, who escape the errors into which he fell, avoid the labyrinths through which he wandered, in which he was often lost in his efforts to find that truth which should have come to him gently, almost unconsciously, even as the gentle rain to parched and thirsty ground.

There are additional reasons why at the end of the nineteenth century the fight for truth for the spread of pure Homœopathy is still to be made, and I shall refer to them briefly. But to my mind the causes already spoken of are quite sufficient. Ignorance or dishonesty, or both, on the part of teachers produces a like crop among those who would and should have been taught, and in a large and ever increasing proportion.

Homœopathy will make converts of all who bring to the study of her claims a clear, unbiased, honest mind, but to secure such students, they must first be convinced of our honesty—honesty of our faith and of our methods. The absence of this assurance, the dishonest grafting of allopathic expedients upon so-called homœopathic practice, has prevented thousands from entering upon the study and examination of our law, our philosophy, our claims, which in a large percentage of such examiners would have resulted in their conversion to the truth. For long, long years our prospects have been dark, the clouds lowering above our Zion, but in the last few years the sun of hope for the future of our school has begun to shine, feebly at first, but gradually growing stronger, in the establishment of homœopathic colleges, not alone in name, but in reality, first *one*, now *three*, where the truth, the *whole* truth, and nothing but the truth, as taught by Hahnemann, his immediate followers, and such of later date on whom the mantles of the above named have fallen.

Already can the good work done in these colleges by these Hahnemannian homœopaths be seen. Already are the graduates from these institutions, knowing what Homœopathy is —for what it should and must stand—knowing our law of cure and how to apply it, are foci from which are beginning to spread among some of our ignorant or careless, aspirations to know and practice Homœopathy as it should and must be known and practiced, in order to be worthy of the name. Perhaps my remarks as to our colleges and their Faculties were too sweeping. In some colleges and Faculties would be found a man who would try to teach Homœopathy in its fulness and purity, to stem the tide of eclecticism which like a flood prevailed. What great and lasting good could result from such a man's efforts and teachings, when by the balance of the Faculty they were laughed at and denied—a double denial in word and practice? But I would be the last to assail any who having done, having tried to do, his duty, yet found not success following his efforts.

Oh! that our colleges, past and present, all of them, had done their whole duty. Then would our school have ere this conquered the errors of superstition and experimentalism in medicine; then would songs of joy and thankfulness be heard in thousands of homes throughout the world over sufferings assuaged and death disappointed, where now hearts and lips are paralyzed by sorrow and loss.

But as I said before or intimated, the dawn breaks, the sun begins to shine, and hope, no longer confined to Pandora's box, begins to animate the hearts, souls, and lives of the disciples of Hahnemann.

Perhaps I might appropriately leave my subject here, but there is another matter demanding notice as it has in the past, does now, and may in the future delay the coming of the day for which we long and look—the day in which our people shall go up and possess the land.

Early in my remarks I referred to professional selfishness, born in us as men, increased in us as physicians by the superstitious reverence of the masses for the possessor of a something, a power they cannot understand, grounded in us by the competitive system under which all business is transacted, intensified by our close corporations, medical councils, and special privileges under the law. To escape the effects which naturally follow these conditions, necessitates frequent personal "stock-taking," the keeping of our failures constantly in mind.

Dogmatism, egotism, superciliousness, medical pharisaism, all offshoots of the professional selfishness mentioned above have been found altogether too commonly among us and not confined to either division in our ranks.

Indulgence therein has prevented progress by accentuating differences, developing wrath, obstinacy, and a disposition to call hard names. Now it being admitted that these evil mental conditions have existed and do exist, and to the injury of our school, it is of no importance to consider to what extent they have obtained, nor the amount of injury done, but rather that we avoid cause and effect in this respect hereafter.

In this connection I may say that I believe the best friends of pure Homœopathy have unintentionally retarded the coming of the day for which they longed and looked, have proved obstacles in the way of the spread of the truth by too great an ostracism of those whose knowledge was limited, and as a consequence whose practice was far from Hahnemannian—by means of strong language and harsh names. The line of cleavage became deeper and wider and more and more distinct, until we have experienced to a very considerable degree the fate of the house divided against itself. Kind words and persuasion produce more lasting good than vituperation and force. A harsh, unkind name is apt to induce a retort in kind, only more so. To my mind the Hahnemannian from the heights he has attained can well afford to extend a helping hand and continue to do so until unnecessary because all know and practice the truth. If we cannot influence and educate so-called homœopaths, how can we expect to do likewise with even greater sinners. The absence of this feeling of gratitude, that "freely have I received, freely will I give," not only exerts an injurious influence not alone upon those on the outside, but also among ourselves, our immediate brethren, tending to cultivate intolerance and illiberality, and it is so easy, so natural to become so affected. I have often thought that Dunham's expression regarding "liberty in homœopathic practice" had its origin in a dread of this very illiberality, this intolerance which he must have felt and noticed was developing among the comparatively few representatives of Homœopathy to be found in his day. An outcry has been heard against him, living and dead, for this utterance, and had it not been impossible to successfully attack his knowledge, teachings, writings, and practice of pure Homœopathy, this spirit of dogmatism and intolerance would have been certain to ostracize him had his useful and all too short life been prolonged, and all because he uttered a plea for liberty! To have liberty, to be truly liberal, we must be within the law. Liberty outside the law becomes license. But even so, before condemning, let us be sure that we have grasped a full knowledge of that law.

Would that all who call themselves homœopaths possessed a like liberality to Dunham, with a like adherence to our law, in life, words, and practice. Having our law of cure and the knowledge and means to apply it which we all possess, we should cultivate charity and humility, until, at least, we never fail to cure all curable cases which come under our care.

Even in societies like our own, composed of men who not only know the truth but to the very best of their ability practice it, this spirit of suspicion and illiberality may obtain. Then, if admitted, it will make no difference that the life, views, and practice of a member have for long years been known to and endorsed by his fellow-members. Let him attempt to take a step in advance, attempt to prove that the law as laid down by Hahnemann is capable of expansion; to prove that where the end of a good, a long, and well utilized life found Hahnemann and the school he founded, should not *necessarily* be the end; to follow Hahnemann's own explicit advice, nay, command, "to test and examine all things," we are apt to at once suspect and accuse him of violations of the law, and, as we accuse the dominant school in their treatment of our own—condemn without proof, without testing. I am not going to bore you with a discussion upon Homœopathy vs. Isopathy, nor upon the misnamed "antidotal method" (the christening of which is but another instance of the coming of an "enemy who sowed tares"), but to plead for more charity in our dealings the one with the other, in our words the one to the other. To plead that we prove our faith, not alone by words, but by deeds, let us by our acts and words, by our spirit, prove our faith in the law under which we practice, assured that if a law of nature, then the world (medical) in arms cannot prevail to alter or prove untrue.

On the other hand, should continued study and examination prove it to be more extensive and far-reaching in its beneficence than even Hahnemann saw it, it is our duty and should be our pleasure to publish the fact, and adopting, spread still further the blessing *similia similibus curantur* has conferred.

Let us remember that it is the letter that killeth, while the spirit maketh alive. In our dealings with one another we may, with advantage, adopt our rules in prescribing and first being sure that a prescription is needed, administer our

*criticism* in a single dose and a high potency, grant plenty of time to permit of its full action, and never repeat the dose without taking the case *carefully* and *correctly*.

Each and all doing his duty as far as in him lies—living up to his opportunities and ever striving to increase them, it needs no prophet to predict that the inherent vitality in our system and school will throw off the *miasms* which have dwarfed growth and limited ability to bless, and Homœopathy will become what it should have been ere this, "the joy of the whole earth."

